# Single breathhold, three-dimensional measurement of left atrial volume and function using sparse CINE CMR imaging with iterative reconstruction

**DOI:** 10.1186/1532-429X-17-S1-Q35

**Published:** 2015-02-03

**Authors:** Pierre Monney, Orestis Vardoulis, Davide Piccini, Amit Bermano, Amir Vaxman, Craig Gotsman, Janine Schwitter, Michael O Zenge, Michaela Schmidt, Mariappan S Nadar, Matthias Stuber, Nikolaos Stergiopulos, Juerg Schwitter

**Affiliations:** 1 Center for Cardiac Magnetic Resonance, Cardiology, University Hospital Lausanne (CHUV), Lausanne, Switzerland; 2 Laboratory of Hemodynamics and Cardiovascular Technology, Swiss Federal Institute of Technology, Lausanne, Switzerland; 3 Center for Biomedical Imaging (CIBM), University of Lausanne, Lausanne, Switzerland; 4 Advanced Clinical Imaging Technology, Siemens Healthcare, Lausanne, Switzerland; 5 Center for Graphics and Geometric Computing, Technion, Haifa, Israel; 6 Geometric Modelling and Industrial Geometry, Vienna University of Technology, Vienna, Austria; 7 Healthcare sector, Siemens AG, Erlangen, Germany; 8 Imaging and Computer Vision, Siemens Corporation, Princeton, NJ, USA; 9 Radiology, University Hospital Lausanne, Lausanne, Switzerland; 10 University of Fribourg, Fribourg, Switzerland

## Background

Left atrial (LA) dilatation is associated with a large variety of cardiac diseases and many LA pathologies go along with an adverse prognosis. A time-efficient 3D CMR method to precisely measure the LA volumes and function is therefore highly desirable.

## Methods

A highly accelerated prototype cine sequence with sparse sampling and Iterative Reconstruction (sCINE-IR) was used in phantoms and patients to acquire 5 cine slices (2 long axis, LAX and 3 short axis, SAX) through the LA during a single breathhold yielding a spatial/temporal resolution of 1.5mm/30ms (1.5T Aera, Siemens AG, Germany). The LA volumes were reconstructed from these 5 slices using a non-model based method (Bermano A, ACM trans Graph 2011). As a reference in patients, a self-navigated high-resolution whole-heart 3D dataset (3D-HR) was acquired during mid-diastole, from which the LA volume was segmented. Phantom study. Five LA phantoms made of solanum tuberosum L of known volume (water displacement method) and of different shapes were imaged with both 3D-HR and CS in various slice orientations and the calculated volumes were compared. Patients study. Three patients were scanned with both 3D-HR and sCINE-IR. The volumes obtained with 3D-HR and with sCINE-IR during the corresponding mid-diastolic frame were compared using Bland-Altman method and linear regression.

## Results

Phantom study. Volumes measured by sCINE-IR were highly correlated with the true LA volume, with a mean difference of -4.7±1.8ml (8.7% underestimation, R^2^=0.94). The calculated volumes were not significantly different when different orientations of the sCINE-IR slices were planned (LAX aligned vs not aligned with the true LA long axis, SAX parallel vs not parallel to the mitral plane, p=ns for both). The mean difference between 3D-HR and true LA volume was -1.4±1.4ml (2.3% underestimation, R^2^=0.97). Patients study. Reference LA volumes were obtained with 3D-HR in 3 patients aged 23-80 years (63ml, 62ml and 395ml). sCINE-IR -calculated volumes of the mid-diastolic frame matched closely the reference volume with a difference of 2.7±6.5ml (2.7% underestimation, R^2^=0.99). Complete time-volume curves of the LAs were obtained for each patient, allowing to assess LA phasic function (Figure).

**Figure 1:**
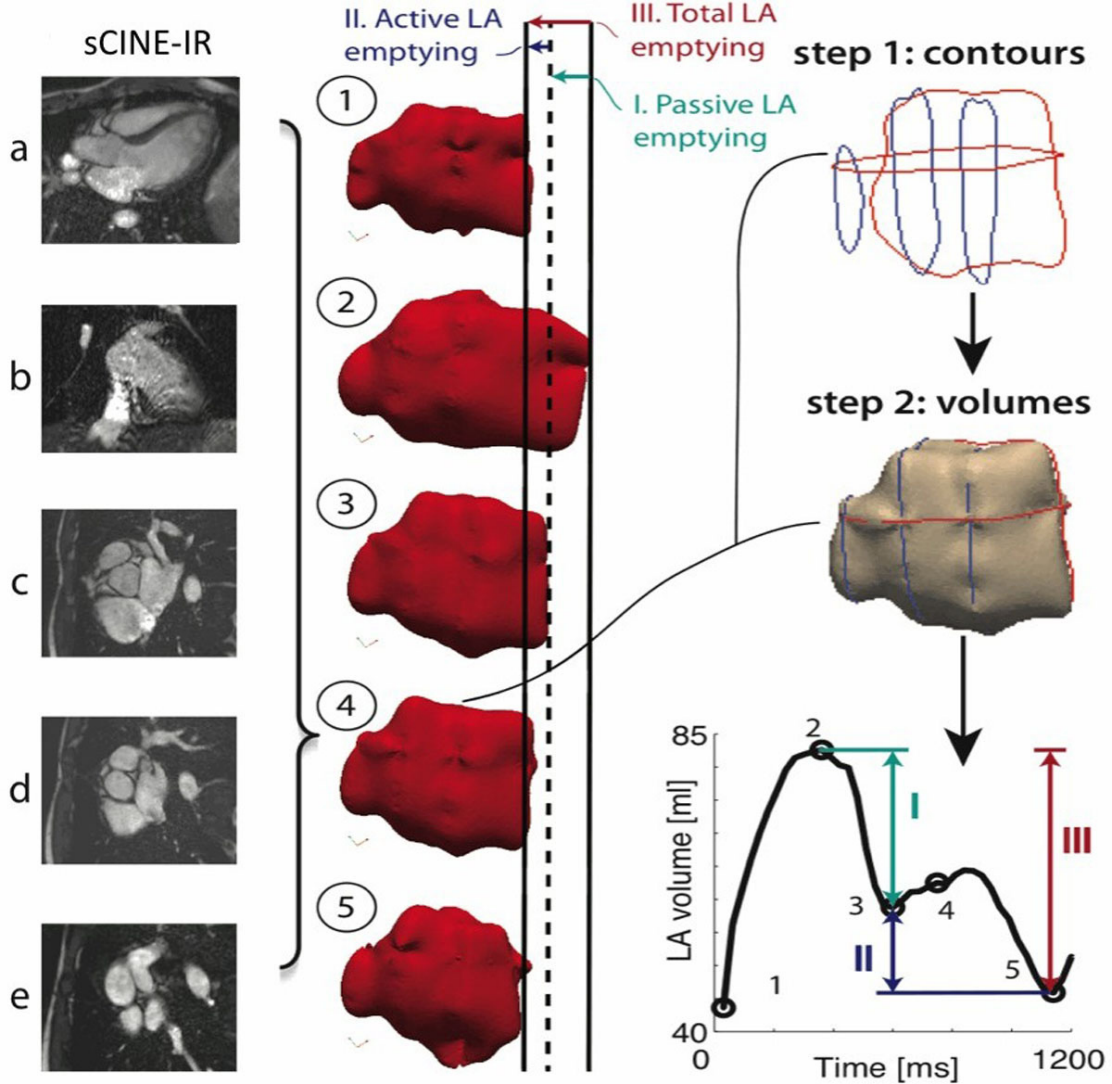
LA volume reconstruction using a sCINE-IR acquisition. Left : sCINE-IR acquisitions through the LA. Images a and b correspond to SAX and images c,d and e to SAX. Middle : the 3D-reconstructed LA volumes (red casts) are shown at 5 different time points during the cardiac cycle, oriented perpendicular to the mitral valve to illustrate the mitral valve motion and lengthening and shortining of the LA long axis. Right : figure at the top illustrates the 5 contours at time point 4, used to reconstruct the LA volume at time point 4 (center right). At lower right, the LA volume curve is given over the entire cardiac cycle (I=LA passive emptying volume; II=LA active emptying volume; III=LA total reservoir volume)

## Conclusions

With this new method of a highly accelerated sCINE-IR acquisition followed by a 3D non-model based reconstruction, LA volumes could be accurately measured from 5 cine slices acquired during one single breath hold. This method opens the possibility to precisely measure LA function over time. The reproducibility of this new technique needs to be assessed on a larger patient cohort.

## Funding

None.

